# Post-traumatic Stress Disorder in Prisoners’ Offspring: A Systematic Review and Meta-analysis

**DOI:** 10.2174/1745017902016010036

**Published:** 2020-04-01

**Authors:** Giacomo Gualtieri, Fabio Ferretti, Alessandra Masti, Andrea Pozza, Anna Coluccia

**Affiliations:** 1 Legal Medicine Unit, Santa Maria Alle Scotte University Hospital of Siena, Siena, Italy; 2 Department of Medical Sciences, Surgery and Neurosciences, University of Siena, Siena, Italy

**Keywords:** Post-traumatic stress disorder, Prisoners, Caregivers, Trauma, Prison, Offspring, Criminal justice system

## Abstract

**Background:**

Parental incarceration can produce serious effects on the offspring’s mental health. The presence of Post-Traumatic Stress Disorder (PTSD) in prisoners’ offspring is understudied and the few literature data showed heterogeneous evidence, with some studies suggesting that about 25% of prisoners’ offspring have PTSD and other reporting much lower prevalence rates around 2-3%. There is no systematic review and meta-analysis about PTSD in prisoners’ offspring.

**Objectives:**

The present systematic review and meta-analysis aimed to provide a first quantitative synthesis of the prevalence of the PTSD diagnosis in prisoners’ offspring. Moderator variables of the effect sizes were assessed, including offspring’s and parents’ gender, offspring’s generational cohort (children/adolescents versus adults), reasons for parental incarceration (political/war versus crime), and country type (Western versus Non-Western countries).

**Methods:**

A systematic review and a meta-analysis were conducted according to the PRISMA guidelines. Studies were included if they assessed the presence of a PTSD diagnosis in child, adolescent or adult offspring of prisoners through a diagnostic classification system, a clinician-administered interview or a self-report questionnaire, if they reported data necessary to calculate the effect sizes or the authors were available to provide them. Studies might have been based upon any design except review, single-case, case series, and case reports. Outcomes might have been measured at any time after parental incarceration. Parental imprisonment was defined as any kind of custodial confinement of a parent by the criminal justice system, including being held as a prisoner of war or for political reasons.

Independent reviewers searched published/unpublished studies through electronic databases and additional sources and extracted the data. A random-effect meta-analysis was carried out by calculating the effect sizes as event rates. Heterogeneity was examined by the *I^2^* and the *Q* statistics. Moderators were assessed through meta-regressions.

**Results:**

Six studies (2512 participants) were included. Fifteen percent of prisoners’ offspring had PTSD, as shown by a significant mean effect size of 0.14 without evidence of publication bias (*95% CI*: 0.081 – 0.249, *p*< 0.001). There were no significant differences on the mean effect sizes between the studies on adults and those on children/adolescents [*Q_(1)_* = 0.00, *p* = .999], between the studies on parents incarcerated for political/war reasons and those for crime [*Q_(1)_* = 0.00, *p* = .979], and between the studies conducted in Western and non-Western countries [*Q_(1)_* = 0.854, *p* = .355]. While offspring’s gender was not related to the effect sizes [*β* = -0.01, *95% CI*: -0.02 – 0.02, *p* = .452], parents’ gender was significantly and positively associated with the effect sizes suggesting that in studies with higher percentages of incarcerated mothers, the prevalence of offspring’s PTSD was higher [*β* = 0.01, *95% CI*: 0.0 – 0.01, *p* = .019].

**Conclusion:**

PTSD is a serious mental health condition among prisoners’ offspring, particularly when mothers are incarcerated. The present findings point out the importance of thorough assessment and timely intervention/prevention strategies implemented by professionals of mental health settings and detention systems. The cross-sectional design of the studies does not allow causal conclusions to be drawn about the effect of parental incarceration as a risk factor for PTSD. Other variables related to parental incarceration may explain these findings. This limitation points out the importance of further longitudinal research.

## 1 INTRODUCTION

Parental incarceration can produce serious psychological effects on offspring and is often related to more adverse offspring’s mental health outcomes than other types of parental separation (*e.g.*, parental death, hospitalization, disharmony) [1-3]. In a literature review, Murray and Farrington [4] concluded that parental imprisonment is probably associated with at least twice the risk of mental health problems for offspring. Specifically, parental imprisonment is associated with a number of psychiatric and psycho-social problems, including Attention Deficit Hyperactivity Disorder, anxiety and depressive disorders, and antisocial delinquent behaviour [5, 6].

Post-Traumatic Stress Disorder (PTSD) is one of the most common psychological reactions to a life-threatening event [7-9]. This condition includes specific symptom clusters, typically arising within the first three months since the event occurrence: (a) re-experiencing symptoms of the event, by means of nightmares, flashbacks, and intrusive memories, (b) avoidance of reminders of the event, (c) hyperarousal symptoms (hypervigilance, impaired concentration, increase in a startle response, and anger outbreak) [10]. Various psycho-social processes may explain why prisoners’ offspring are at higher risk of developing PTSD than those of the general population, such as enduring loss of or separation from parents (especially when unexpected or unexplained), feelings of not being loved by the parent, experiencing the psychopathological conditions of the imprisoned parent (*e.g.*, alcohol/substance abuse) or maladaptive psychological reactions reported by the other parent, exposure to the parent's criminal activity, witnessing the parent's arrest and court proceedings, loss of family income, housing instability, changes in caregiving, stressful visits with the incarcerated parent, perceived stigma, and the characteristics of the prisons’ environment (*i.e*., child-friendly features such as rules regarding visits/telephone contacts) [11].

In Western countries, about 0.5-2.3% have a parent residing in prison [12, 13]. The presence of PTSD in the offspring of prisoners is, however, an understudied research topic and under-recognized problem by practitioners. The few existing studies indicated that PTSD can be a serious mental health condition among prisoners’ offspring. However, the literature data showed heterogeneous evidence, with some studies suggesting that about 25% of prisoners’ offsprings have a PTSD diagnosis [14, 15] whereas other studies report much lower prevalence rates, around 2-3% [16, 17]. Currently, there is no systematic review and meta-analysis about PTSD in prisoners’ offspring.

The present paper describes the first systematic review and meta-analysis aimed to provide a quantitative synthesis on the prevalence rates of PTSD diagnosis in prisoners’ offspring. Specific moderator variables of the mean effect sizes were assessed, including offspring’s gender, parents’ gender, offspring’s generational cohort (comparing studies conducted on children/adolescents and those conducted on adults), reasons for parental incarceration (political/war versus crime reasons), and type of country [Western (Anglophone and European countries) or Non-Western countries (African, Middle-Eastern, Central and Southern America, Asian countries)].

## 2 METHODS

### 2.1 Eligibility Criteria

The protocol of the review was prepared following the PRISMA-Protocol guidelines (PRISMA-P) [18] and it can be provided by the corresponding author on request.

For the present review, parental imprisonment was conceptualized as any kind of custodial confinement of a parent by the criminal justice system, including being held as a prisoner of war or for political reasons. Studies were included if they met the following criteria: (a) they assessed the presence of a PTSD diagnosis in offspring of prisoners, (b) they reported data necessary to calculate the effect sizes on the prevalence of a diagnosis of PTSD (total sample sizes and the number of participants with PTSD) or study authors were available to provide these data if they were not reported in the paper, (c) the diagnosis of PTSD was based on an official diagnostic classification system (*i.e*., any version of the DSM or ICD) and at least one structured/semi-structured diagnostic, clinician-administered interview [*e.g.*, Clinician-Administered PTSD Scale (CAPS; [19]) or a self-report questionnaire, (d) the paper was published in English, French, German, Spanish or Italian language. Studies that were considered for inclusion could use any research design except reviews, single-case studies, case series, and case reports. Eligible designs included cross-sectional or case-control studies where the prevalence of PTSD was assessed in prisoners’ offspring. Longitudinal studies were included if they reported at baseline data regarding the prevalence of PTSD in this type of population or the authors were available to provide them if requested. Outcomes might have been measured any time after parental incarceration: while parents are in prison or after release, in childhood or in adulthood. Studies were included if they were conducted on child, adolescent or adult offspring.

### 2.2 Information Sources and Search Procedure

Studies were identified by conducting an online systematic search of electronic databases and by using keywords related to PTSD (“Post-traumatic stress disorder” OR“Traumatization” OR “Trauma”) combined through the Boolean operator AND with keywords related to prisoners (“Prisoners” OR “Incarceration” OR “Pow” OR “Captivity”) and with keywords related to offspring (“Offspring” OR“ Sons” OR“Children”). The search procedure was conducted during the second week of November 2019 by using the databases Scopus and PubMed. No date restriction was applied.

In addition, all corresponding authors of the eligible studies were contacted to identify any further studies, irrespective of their publication status. Reference lists of the studies included in the meta-analysis were also examined.

### 2.3 Selection of Studies

Studies were screened against eligibility criteria by two authors (AP, FF) working independently in three stages. During the first and second stages, studies were examined with regards to the inclusion criteria after reading the title and the abstract, respectively. After each stage, the authors met to compare their selections. Studies were not excluded if there was disagreement between the authors on inclusion or exclusion. During the final stage, two authors examined independently the full text of the papers. At this stage, any disagreements about inclusion or exclusion of studies were discussed and resolved in a meeting with a third author (AC).

### 2.4 Data Extraction and Coding

All the information was extracted from each included study by two authors (AP, FF) working independently and inserted into an Excel worksheet, which was piloted first on 2 included studies. The following information was extracted and coded from each study: (1) Title of the paper, (2) First author, (3) Publication date, (4) Country where the study was conducted, (5) Research design, (6) Recruitment setting (*i.e*., sites where participants were recruited and strategies used to recruit them), (7) Inclusion and exclusion criteria, (8) Total sample size, (9) Group size with PTSD, (10) Number of offspring without PTSD, (11) Mean age of the offspring total group, (12) Offspring generational cohort (coded as children/adolescents versus adults), (13) Total percentage of female offspring, (14) Instrument(s) used to make the PTSD diagnosis, (15) Total percentage of mothers, (16) Reasons for parental incarceration (war/political reasons versus crime reasons).

A third author (AC), not involved in the data extraction procedure, checked the correctness of the data entered in the worksheet. After entering the data, any discrepancies were discussed at a meeting between the authors who extracted the data and the third author.

### 2.5 Meta-analytic Procedure

A random-effect meta-analysis was conducted using the software Comprehensive Meta-Analysis, CMA version 2.00 [20]. The effect sizes were calculated as event rates, which were obtained as the ratio between the number of cases with PTSD and the total study sample size: higher effect sizes indicated higher prevalence rates of PTSD in offspring samples. The effect sizes were estimated by adopting a 95% confidence interval. Hedges’ correction for small sample bias was applied to all effect sizes [21].

Heterogeneity analysis of the effect sizes was conducted by calculating the *I^2^* statistic [22] and the *Q* index [21]. The *I^2^* index represents a measure of between-study heterogeneity in percentage, which is attributable to variability rather than chance [22]. A value approximating zero indicates homo-geneity, whereas values of 25%-50%, 50%-75%, and 75%-100% represent low, medium, and large heterogeneity, respectively. The *Q* index is calculated by summing the squared deviations of each study’s effect estimate from the overall effect estimate, while weighting the contribution of each study by its inverse variance [21].

To assess the likelihood that the effect sizes were subjected to publication bias, two procedures were used: the visual inspection of the funnel plot and the Egger test [20]. The first represents a scatter plot in which the effect sizes computed from the included studies are plotted on the horizontal axis against an indicator of study precision, the standardized error, on the vertical axis [22]. In the absence of bias, the graph resembles a symmetrical inverted funnel, because the effect sizes derived from smaller studies scatter more widely at the bottom of the graph, with the spread narrowing with increasing precision among larger studies. If there is publication bias because smaller studies that report no significant effect sizes remain unpublished, the funnel plot appears asymmetrical [22]. The Egger test is an unweighted regression analysis based on the precision of each study as the independent variable and the effect size divided by its standard error as the dependent variable [23]. A non-statistically significant result of the t-test for the null hypothesis of an intercept equal to zero allows us to discard publication bias [23].

Three sensitivity analyses were performed calculating the effect sizes in (a) the studies including only adults or in those including only children/adolescents; (b) the studies conducted on offspring of parents incarcerated for political/war reasons or those incarcerated for crime; (c) the studies conducted in Western (Anglophone and European Union countries) or Non-Western countries (African, Middle-East, Central and Southern America, Asian countries).

## 3 RESULTS

### 3.1 Selection of the Studies

**Fig. (1):**
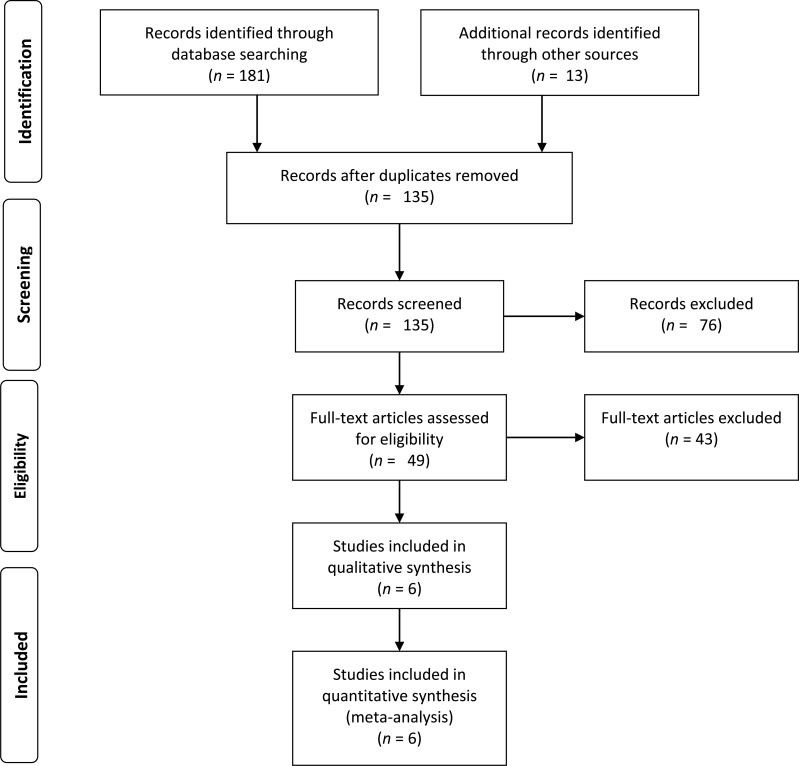
PRISMA flowchart of the study selection.

The search through the databases and the additional sources produced a total of 181 records, assessed independently in the first phase by two authors. After duplicates were removed, 135 records were assessed *via* title/abstracts. This led to the exclusion of 76 records. Reviewing the full text of the remaining 49 records resulted in the exclusion of 43 articles. After this selection process, 6 studies (2512 participants overall) were included in the systematic review and meta-analysis. The PRISMA flowchart of the study selection process is provided in (Fig. **1**).

### 3.2 Descriptive Characteristics of the Studies

The sample sizes in the included studies ranged from 79 to 1869 participants. Three studies were conducted in the United States, one in Israel, one in the State of Palestine, one in Lithuania. Three studies were conducted on adults, 3 studies on children. Two studies were conducted on the offspring of parents incarcerated for crime reasons and four studies conducted on the offspring of parents incarcerated for political/war reasons. All the studies were based upon a cross-sectional research design, except a longitudinal one [17]. All the papers were written in English and published in peer-review journals. The descriptive characteristics of the included studies are presented in Table **1**.

### 3.3 Meta-analysis on the Prevalence of PTSD in Prisoners’ Offspring

**Fig. (2):**
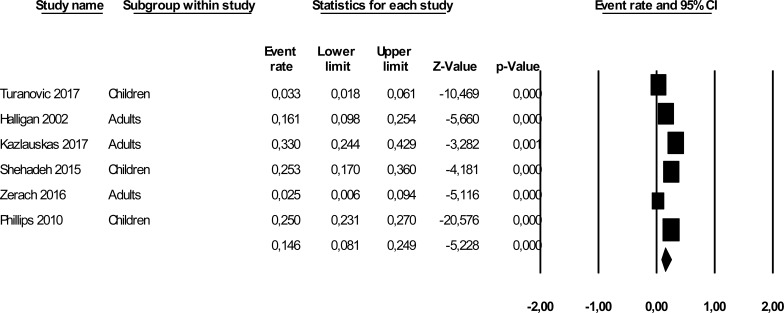
Forest plot of effect sizes.

**Fig. (3):**
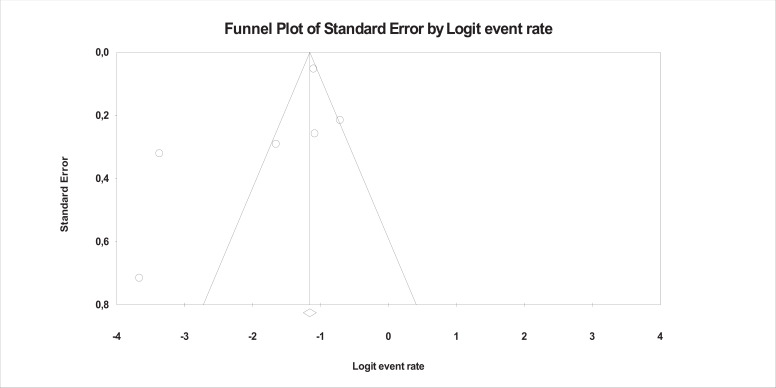
Funnel plot of publication bias.

The mean effect size was statistically significant and showed that about 15% of the offspring of prisoners had PTSD (Event Rate = 0.146, *95% CI*: 0.081 - 0249, *Z* = -5.228, *p*< 0.001, *k* = 6). The forest plot with study and mean effect sizes is provided in Fig. **(2)**. There was no evidence of publication bias, as suggested by the funnel plot (Fig. **3**) and by the Egger test of intercept which was not statistically significant[*β* = -2.853,*SE* = 1.909, *t*_(4)_ = 1.494, *p* = 0.209]. For this analysis, a significant heterogeneity was found [*I^2^* = 92.637, *Q*_(5)_ = 67.906, *p* < .001].

### 3.4 Sensitivity and Moderator Analysis

**Fig. (4):**
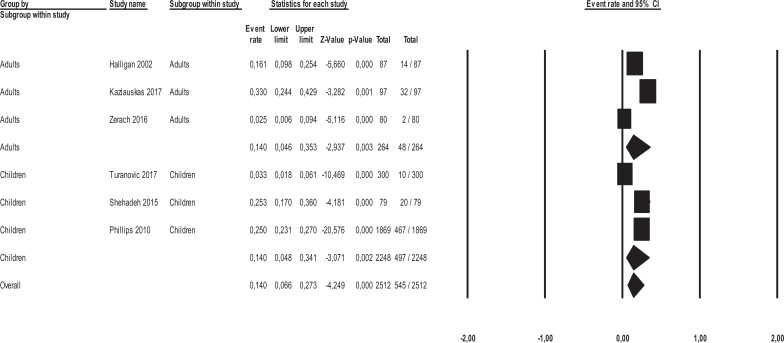
Forest plot of effect sizes across adult and child/adolescent offspring samples.

**Fig. (5):**
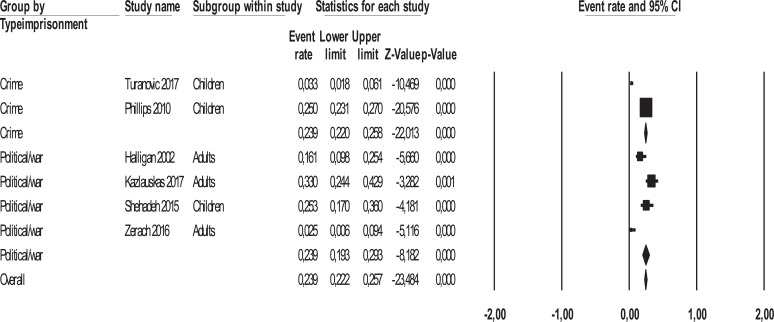
Forest plot of effect sizes of studies on parents incarcerated for political/war reasons and crime.

**Fig. (6):**
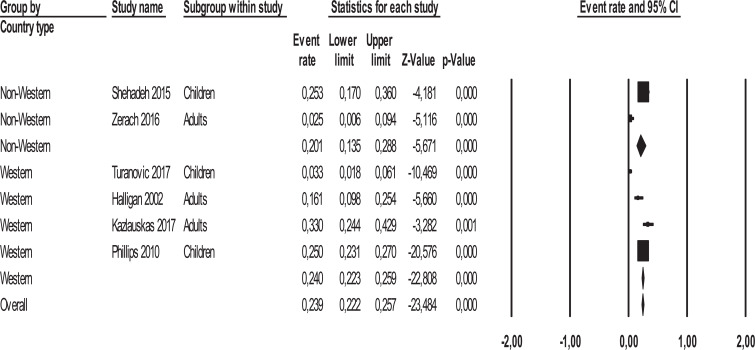
Forest plot of effect sizes for studies conducted in Western and Non-Western countries.

Three sensitivity analyses were carried out by calculating the mean effect size separately in (a) adult samples and child/adolescent samples, (b) in the studies conducted on offspring of parents incarcerated for political/war reasons and for crime reasons, and (c) in studies conducted in Western and non-Western countries. There was no significant difference in the mean effect sizes between the studies on adults and those on children/adolescents [*Q*_(1)_ = 0.00, *p*= .999] (Fig. **4**). No significant differences in the mean effect sizes were found between the studies conducted on the offsprings of parents incarcerated for political/war reasons and those for the crime [*Q*_(1)_ = 0.00, *p* = .979] (Fig. **5**) and between the studies conducted in Western and non-Western countries [*Q*_(1)_ = 0.854, *p* = .355] (Fig. **6**).

Subsequently, offspring’s gender and parents’ gender, coded as the percentage of female offspring and as the percentage of mothers, respectively, were assessed by running simple meta-regressions. While offspring’s gender was not related to the effect sizes [*β* = -0.01, *95% CI*: -0.02 – 0.02, *p* = .452, *k* = 5], parents’ gender was significantly and positively associated with the effect sizes, suggesting that for the studies with higher percentages of incarcerated mothers, the prevalence of PTSD among the offspring was higher [*β* = 0.01, *95% CI*: 0.0 – 0.01, *p* = .019, *k* = 5].

## 4 DISCUSSION

PTSD is a serious mental health condition which appears not sufficiently studied in prisoners’ offspring. The present paper describes the results of the first systematic review and meta-analysis which assessed the prevalence of a PTSD diagnosis in prisoners’ offspring and investigated the role of specific moderator variables such as offspring’s and parents’ gender, offspring’s generational cohort, reasons for parental incarceration, and type of country.

Overall, about 15% of the prisoners’ offspring had a PTSD diagnosis without consistent evidence of publication bias. On the one hand, this prevalence rate is higher than the rates of the full PTSD diagnosis (1.7%) and the subthreshold diagnosis (8.8%) observed in adult samples exposed to any other kind of trauma [26]; on the other hand, it is comparable with the prevalence rates of PTSD in children samples exposed to any other kind of trauma [27]. This prevalence rate was, however, lower than the data (25%) reported by studies on other types of traumatic events related to parental separation, such as a previously published study on children who had experienced recent and sudden parental death [28].

Further analyses showed some heterogeneity across the studies suggesting the need for investigating the role of potential moderator variables. Prevalence of PTSD did not seem to be different across child/adolescent and adult samples, across offspring of parents incarcerated for political/war reasons and for crime, and across Western and non-Western countries. It may be hypothesized that the reasons for parental incarceration have a less important role than the event of incarceration per se. With regard to the country type, this finding appears in contrast with the evidence on other psychiatric disorders whose prevalence and risk differ across socio-cultural contexts [29]. However, it may be hypothesized that the categorization of Western versus non-Western countries does not detect actual differences in the detention systems and perhaps other variables more closely related to this effect can reflect differences between PTSD prevalence across countries.

In addition, PTSD was not associated with the offspring’s gender. The lack of an association between offspring’s gender and PTSD appears in line with recent evidence provided by primary studies and reviews where gender was not a predictor of an increased risk of this psychiatric condition [10, 30-32]. However, in another primary study [33] investigating only PTSD symptoms, sons were found to report more severe symptoms than daughters, particularly numbing symptoms. This discrepancy suggests the need for further studies exploring gender differences on symptom clusters, instead of considering the full diagnosis of PTSD.

Interestingly, we found that maternal incarceration was associated with a higher prevalence of PTSD among offsprings. These results may be considered in line with the theoretical conceptualizations and empirical data found in victims with PTSD of life-threatening events showing that maternal separation is a serious risk factor for the development of PTSD [32-34].

In conclusion, our review sheds some light on the association between parental incarceration and PTSD in prisoners’offspring and suggests additional points which need to be investigated due to some limitations of our study.

### 4.1 Limitations and Future Directions

The relatively small number of studies highlights the importance of future studies on this topic and suggests that it is still neglected by researchers. The cross-sectional design of the included studies did not allow us to draw firm conclusions about the effect of parental incarceration as a risk factor for the development of PTSD. Other variables may explain the prevalence rates and may amplify the effects of incarceration. The role of further moderators should be assessed such as the quality of parent– child relationships before the incarceration, pre-existing life difficulties (*e.g.*, early childhood trauma or prenatal exposure to drugs and alcohol), time since trauma occurrence (duration of untreated illness), child’s age at the time of incarceration, nature and length of the sentence, alternative care arrangements, number/quality of the contact with the incarcerated parent, parents’ psychiatric disorders and types of detention systems, or protective factors such as how other family members cope with the event and the wider social context [35-37]. The lack of control groups prevented us from comparing the prevalence rates of PTSD in prisoners’ offspring with those reported in children recruited from the general population. In addition, it may be interesting to compare the prevalence rates with those of children who have experienced the separation from parents for other reasons than incarceration. Another research gap regards the under-representation of European countries. Future research should investigate which PTSD symptom clusters are more severe among prisoners’ children. Finally, new investigations should focus on concurrent psychiatric conditions associated with PTSD in youth such as severe social withdrawal.

In conclusion, the present paper describes the first meta-analysis on the prevalence of PTSD among prisoners’ offspring. This psychiatric condition can be a serious mental health problem in this population, particularly for the offspring of incarcerated mothers. Our findings point out the importance of thorough assessment and timely intervention/prevention strategies implemented by professionals in mental health settings and detention systems.

## CONCLUSION

The present review is the first one in the literature investigating PTSD among prisoners’ offspring. These findings show that it is a serious psychiatric condition, particularly when mothers are incarcerated, suggesting a thorough ass-essment and timely intervention/prevention strategies imp-lemented by professionals of psychiatric settings and detention systems.

## Figures and Tables

**Table 1:** Descriptive characteristics of the included studies (*n*= 6).

**First Author and Year**	**Country**	**Design**	**Recruitment Setting**	**Inclusion Criteria**	**Exclusion Criteria**	**Subjects with PTSD/Tot. Sample (%)**	**Mean Age or Range in Years; Cohort; Females’ Percentage**	**Instruments used to make PTSD diagnosis**
Halligan 2002 [24]	United States	Cross-sectional	Lists obtained from the Jewish commun-ity or responded to community group ann-ouncements and news-paper advertisement. Participants of short-term group psycho-therapy at the Mount Sinai Specialized Tre-atment Program for Holocaust Survivors and their families	Holocaust survivor offspring raised by a parents	Not reported in the paper	14/87 (16%)	42.30; adults; 62%	Clinician Administered PTSD Scale (CAPS; Blake *et al*., 1995)
Kazlauskas 2017 [25]	Lithuania	Cross-sectional	data for this study from a database built up during the research project “Long-term Effects of Political Oppression in Lithuania” which aimed at exploring long-term effects of political violence (1940–1991) in Lithuania, and was conducted by the Vilnius University Trauma Research Group in collaboration with the Lithuanian Genocide and Resistance Research Center (Gailiene & Kazlauskas, 2005	Not reported in the paper	Not reported in the paper	32/110 (29.10%)	44.65; adults; 61.80%	Impact of Event-Scale Revised (IES-R; Weiss & Marmar, 1997)
Phillips 2002 [14]	United States	Cross-sectional	Six agencies in Arkansas and Texas recruited subjects from sequential intakes. These agencies included two not-for-profit child and adolescent mental health provider organizations, mental health clinics operated by two teaching hospitals, a psychiatric hospital, and an adolescent medicine clinic with mental health specialists on staff.	Not reported in the paper	Mental retardation (IQ < 70), psychosis. Adolescents were also excluded if an adult informant who had at least weekly contact with the adolescent during the previous six months was not available to serve as a co-informant.	467/1869 (25%)	13.7, Range = 11-18; adolescents; 42%	Diagnostic Interview Schedule for Children, Present State version (DISC-PS; Shaffer *et al*., 2000)
Shehadeh 2015 [14]	Palestinian State	Cross-sectional	Israeli prisons	Families with children between 3 and 10 years old and when there was more than 1 child in this age range.	Not reported in the paper	20/79 (25%)	7.7; children; 44.2%	UCLA-PTSD-Reaction Index (UCLA-PTSD-RI; Rodriguez *et al*., 1999)
Turanovic 2017 [16]	United States	Cross-sectional	Arizona Department of Corrections	Incarcerated mothers who reported having at least one minor child	Not reported in the paper	22/700 (3.2%)	Range: 1-17; children; not females’ percentage reported in the paper	Semi-structured interview
Zerach 2016 [17]	Israel	Longitudinal	Adult offspring of veterans of the 1973 Yom Kippur War: adult children of former prisoners of war	Offspring of captured veterans	Not reported in the paper	2/98 (2.7%)	35.19, range = 22-53; adults; 53%	PTSD Inventory (Solomon *et al*., 1993)
